# Aquatic community response to volcanic eruptions on the Ecuadorian Andean flank: evidence from the palaeoecological record

**DOI:** 10.1007/s10933-017-0001-0

**Published:** 2017-10-05

**Authors:** Frazer Matthews-Bird, Stephen J. Brooks, William D. Gosling, Pauline Gulliver, Patricia Mothes, Encarni Montoya

**Affiliations:** 1 School of Environment, Earth and Ecosystem Sciences, The Open University, Walton Hall, Milton Keynes, MK7 6AA UK; 2 Department of Biological Sciences, Florida Institute of Technology, 150 West University Blvd, Melbourne, FL 32901 USA; 3 Life Sciences, Natural History Museum, Cromwell Road, London, SW7 5BD UK; 4 Institute for Biodiversity and Ecosystem Dynamics (IBED), University of Amsterdam, Postbus 94248, 1090 GE Amsterdam, The Netherlands; 5 NERC Radiocarbon Facility (East Kilbride), SUERC, Scottish Enterprise Technology Park, Rankine Avenue, East Kilbride, G75 OQF Scotland, UK; 6 Instituto Geofísico, Escuela Politécnica Nacional, Ladrón de Guevara E11-19 253, Apartado, 1701-2759 Quito, Ecuador; 7 Institute of Earth Sciences Jaume, Almera (ICTJA-CSIC), Sabaris s/n, c/Sole, 08028 Barcelona, Spain

**Keywords:** Chironomids, Lake sediment burial, Long-term changes, Non-Pollen Palynomorphs (NPP), Sensitivity, Tephras

## Abstract

Aquatic ecosystems in the tropical Andes are under increasing pressure from human modification of the landscape (deforestation and dams) and climatic change (increase of extreme events and 1.5 °C on average temperatures are projected for AD 2100). However, the resilience of these ecosystems to perturbations is poorly understood. Here we use a multi-proxy palaeoecological approach to assess the response of aquatic ecosystems to a major mechanism for natural disturbance, volcanic ash deposition. Specifically, we present data from two Neotropical lakes located on the eastern Andean flank of Ecuador. Laguna Pindo (1°27.132′S–78°04.847′W) is a tectonically formed closed basin surrounded by a dense mid-elevation forest, whereas Laguna Baños (0°19.328′S–78°09.175′W) is a glacially formed lake with an inflow and outflow in high Andean Páramo grasslands. In each lake we examined the dynamics of chironomids and other aquatic and semi-aquatic organisms to explore the effect of thick (> 5 cm) volcanic deposits on the aquatic communities in these two systems with different catchment features. In both lakes past volcanic ash deposition was evident from four large tephras dated to c.850 cal year BP (Pindo), and 4600, 3600 and 1500 cal year BP (Baños). Examination of the chironomid and aquatic assemblages before and after the ash depositions revealed no shift in composition at Pindo, but a major change at Baños occurred after the last event around 1500 cal year BP. Chironomids at Baños changed from an assemblage dominated by *Pseudochironomus* and *Polypedilum nubifer*-type to *Cricotopus/Paratrichocladius* type-II, and such a dominance lasted for approximately 380 years. We suggest that, despite potential changes in the water chemistry, the major effect on the chironomid community resulted from the thickness of the tephra being deposited, which acted to shallow the water body beyond a depth threshold. Changes in the aquatic flora and fauna at the base of the trophic chain can promote cascade effects that may deteriorate the ecosystem, especially when already influenced by human activities, such as deforestation and dams, which is frequent in the high Andes.

**Electronic supplementary material:**

The online version of this article (doi:10.1007/s10933-017-0001-0) contains supplementary material, which is available to authorized users.

## Introduction

Explosive volcanic eruptions eject large amounts of silicate rock particles, known as tephra, into the Earth’s atmosphere, which can eventually be deposited within the sediments of aquatic ecosystems. The environmental effects of tephra deposition, particularly on the benthic biota of the aquatic community, are difficult to quantify and are likely location specific (Telford et al. 2004). Modern monitoring studies have identified a detrimental effect of tephra on aquatic ecosystems after a volcanic eruption (Kurenkov 1966; Fazlullin et al. 2000; Collier 2002), however, often these studies do not cover a sufficient time frame to understand the lasting long-term effects on the aquatic communities (Telford et al. 2004). Furthermore, high impact rapid changes to the basal element of lacustrine food chains will have cascading consequences and may extend to the surrounding terrestrial ecosystem (Schulz et al. 2015). The palaeoecological record provides an opportunity to investigate the influence of volcanism on lake ecosystems both temporally, on long timescales (100–1000 years), and geographically across landscapes (10–100 km^2^). A more detailed understanding of the long-term effects of volcanism on aquatic habitats can help inform conservation and mitigation strategies. Here we use the palaeoecological record of two lakes to understand the long-term effects of volcanism in the high Andes, along the eastern Andean flank of Ecuador.

Tephra deposits can cause significant changes to lakes and their catchments (Eicher and Roundefell 1957; Baross et al. 1982; Hickman and Reasoner 1994; Zielinski 2000). The effects of tephra deposition can be both physical and chemical. Tephra will, in the short term, reduce light penetration due to the increased particulates in the water column (Abella 1988). An increase in sedimentation from either particulate settling or tephra erosion after a volcanic event may seal off the sediment–water interface, bury macrophytes, and prevent the recycling of nutrients such as phosphorus (Barker et al. 2000). Changes to lake chemistry can result from increased nutrient input from chemical weathering of tephra, and changes in lake water pH and salinity from siliceous deposition (Haberyan 1998; Telford et al. 2004). Whether physical, chemical, or both, volcanism has the potential to change aquatic ecosystems rapidly and cause regime shifts that could persist for hundreds of years (Hickman and Reasoner 1994; Barker et al. 2000).

Tephra input into a fresh water system is the result of either aeolian deposition or catchment erosion. The quantity of material ejected and its residence time within a catchment may determine the lasting effects of an event (Hickman and Reasoner 1994; Barker et al. 2000). As a result, certain lakes may be more sensitive than others and multiple sites may experience different consequences to similar or even identical events. Indeed, palaeoecological studies have shown a range of responses from aquatic communities to tephra deposition. Hickman and Reasoner (1994), in Canadian alpine lakes, noted a ten-fold increase in diatom concentrations lasting about 300 years after tephra deposition. By contrast other studies infer minor short-term changes, from few years to just several days or even no direct response at all, to tephra deposition (Lotter et al. 1995; Telford and Lamb 1999; Self et al. 2015). The response of a particular lake is difficult to predict and may differ between eruptions. For instance, a river-fed lentic ecosystem with a large catchment may receive a greater initial impact from a volcanic eruption than a closed basin (Telford et al. 2004), as more tephra is available to the former system from erosion into the habitat. On the other hand, river-fed habitats with a constant supply of fresh water may actually recover faster than closed basins as the tephra is not locked in the system and instead can be mobilised downstream or quickly replaced with fresh sediments brought in (Collier 2002). The size of the lake itself will also determine the lasting effect of a volcanic event since the effect of an eruption may be magnified in a small lake, as opposed to a larger setting (Barker et al. 2000).

Chironomidae, a diverse family of two-winged aquatic insects of the order Diptera (commonly known as non-biting midges), have long been used as freshwater quality indicators (Thienemann 1922) and have recently been used for palaeotemperature reconstructions in the tropical Andes (Matthews-Bird et al. 2016a). Chironomids inhabit a wide range of biotopes but most species are aquatic, few fresh water habitats do not support a chironomid population (Armitage et al. 1995). Chironomidae play a critical role within most aquatic food webs, they are a valuable source of food for other organisms, notably fish and birds, and are integral in the cycling of nutrients between primary and secondary consumers (Pinder 1986). Most species have been shown to be stenotopic and this, combined with rapid generational turnover (annual/sub-annual) and a mobile winged adult, means the family are extremely sensitive indicators of environmental change (Porinchu and MacDonald 2003).

Despite volcanism being a major feature of the Andes, little is known about the effects of volcanic activity on watersheds in the region. Here we use sub-fossil chironomid larvae to assess the long-term (centennial) effect of tephra deposition in two Andean flank lakes. The response of the chironomids is compared to the response of other aquatic communities occupying different trophic levels (autotrophs, mainly aquatic and littoral vegetation) from the same sediment sequence. Using the palaeoecological record, we assess the sensitivity and resilience of aquatic ecosystems to tephra deposition and the effects of light suppression, chemistry changes and burying. In addition, the potential influences of catchment features (e.g. openness of the landscape, water bodies’ connectivity) are also considered.

### Study sites

The Andean flank is a highly tectonic region, and experiences significant volcanic activity due to the continued convergence of the Nazca and Antarctic plates with the South American plate (Barberi et al. 1988). The Andean range is split into four volcanic belts: the Northern, Central, Southern and Austral Volcanic Zones. Both lakes analysed in this study lie within the direct influence of the Northern Volcanic Zone (NVZ) that spans northern Ecuador and Colombia (ESM1). Over 20 volcanoes are recognised as active across the NVZ both today and on Quaternary timescales (Hall et al. 2008). In this work, two lakes (Lagunas Pindo and Baños) have been selected based on their specific features: both are located at similar distances to active volcanoes, but greatly differ in the catchment characteristics and the exposure frequency to volcanic activity.

**Fig. 1:**
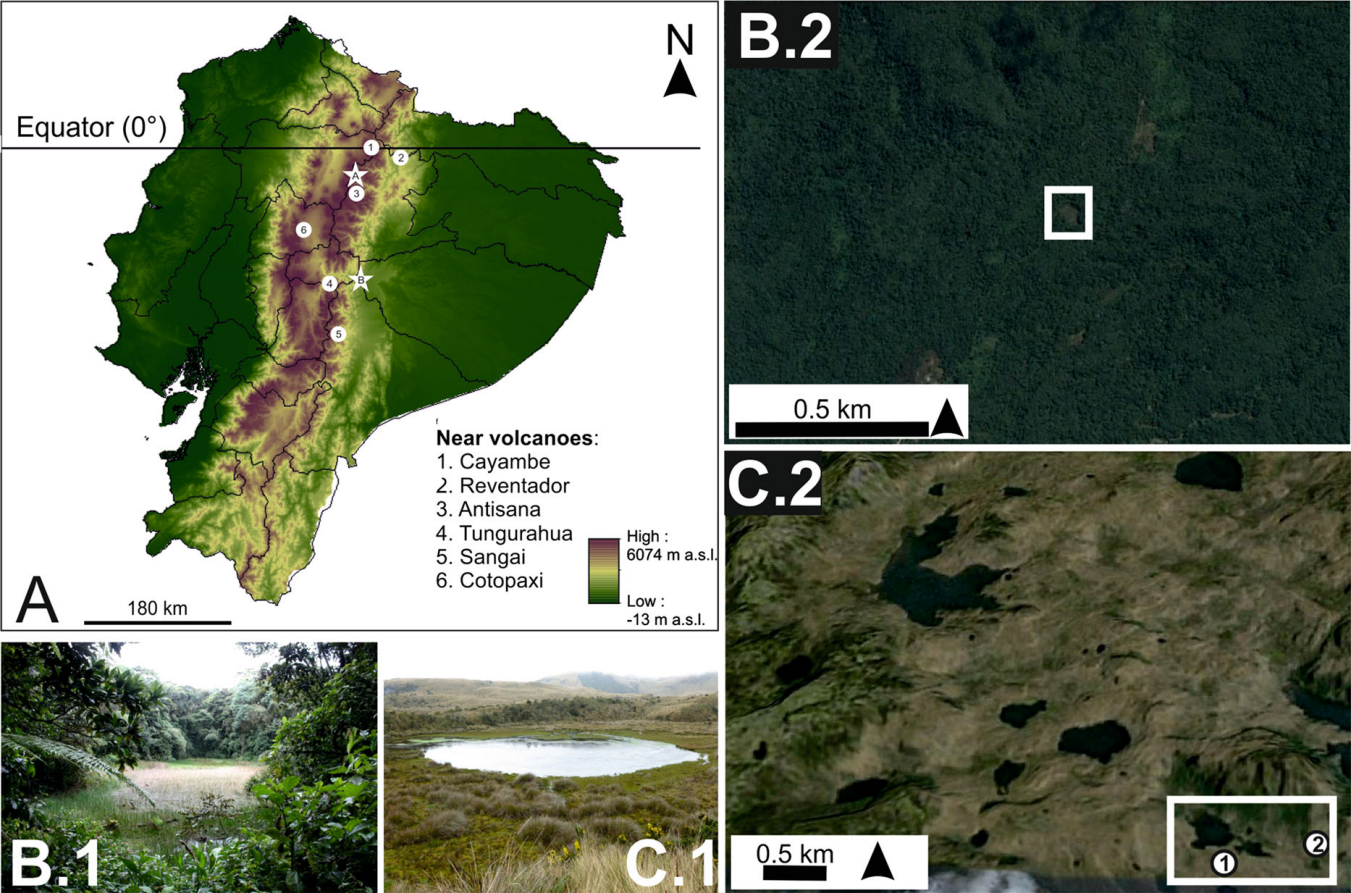
**a** Map of Ecuador, showing location of the lakes studied (marked as stars: **a** Laguna Baños and **b** Laguna Pindo) and the main volcanoes close to the lakes (circles). **b** Laguna Pindo: Image of the lake (b.1), and Google Earth view of the catchment (b.2). **c** Laguna Baños: image of the lake (c.1), and Google Earth view of the catchment (c.2: numbers 1 and 2 indicate the water body analysed in Michelutti et al. (2016) and the present study respectively). Google Earth access on February 2016. Note the differences in the scale used for both images and in the openness of the catchment showed by colours in the online version (green/dark = forest; yellow/light = grassland)

Laguna Pindo is a small shallow lake (1.2 m depth) and roughly circular in shape (around 40 m diameter) on the eastern Andean flank of Ecuador (1°27.132′S–78°04.847′W; Fig. 1). The site is located in Pastaza province near the town of Mera at an elevation of 1248 m a.s.l. Mean annual temperature is about 20 °C with little seasonal variation, annual precipitation can reach up to 4000 mm per year (Hijmans et al. 2005). Currently the lake is not directly fed by an in-flow and has no visible out-flow (Fig. 1); the lake receives water from surface run-off and direct precipitation. Laguna Pindo is positioned in the Andean foothills on a steep slope dropping down to the Pastaza river basin, there are no obvious geomorphological causes for the escarpment of the lake and we hypothesize it is tectonic in origin. The lake is heavily overgrown with aquatic plants (Cyperaceae) and completely vegetated to the water’s edge by a closed forest which surrounds the site with a canopy of 15–25 m high. The site lies within lower montane forest (Harling 1979) and dominant species belong to the families Melastomataceae, Araceae, Urticaceae (members of former Cecropiaceae), Euphorbiaceae, Myrtaceae, Rubiaceae, Myristicaceae, Asteraceae and Mimosoideae (Fabaceae). Lianas, epiphytes (Bromeliaceae, Orchidaceae), and tree ferns are also common. Laguna Pindo is approximately 40 km from Tungurahua, an andesitic-dactic stratovolcano and one of South America’s most active (Fig. 1). Frequent ash explosions producing plumes several kilometres high, as well as pyroclastic flows, characterize Tungurahua (Hall et al. 1999; Le Pennec et al. 2008, 2013).

Laguna Baños is a small glacially formed lake 1 m deep, close to the town of Papallacta in the province of Napo on the eastern flank of the Ecuadorian Andes, situated at an elevation 3821 m a.s.l. (0°19.328′S–78°09.175′W; Fig. 1). The site is in a high lake district region of Cayambé Coca National Park, surrounded by Páramo vegetation (tropical alpine grassland). Mean annual temperature is about 5.7 °C and mean annual precipitation is approx. 1300 mm per year (Hijmans et al. 2005). Although temperatures are almost constant throughout the year, daily variations can be ~ 20 °C (Jørgensen and León-Yánez 1999). The lake is roughly circular (around 55 m in diameter) and is the last in a sequence of three lakes connected by a small stream (Laguna Baños system; Fig. 1). Consequently, the lake receives water through inflow, surface run-off and direct precipitation. The site is currently heavily in-filled by aquatic plant and algal growth with a strong lotic influence. The surrounding vegetation is characteristic of grass Páramo, dominated by *Calamagrostis* sp. and *Festuca* sp. (Poaceae), with scattered patches of shrubby vegetation (Asteraceae) and mixed *Polylepis* sp. (Rosaceae) woodlands. Laguna Baños is about 19 km from the summit of Antisana (Fig. 1), a large stratovolcano, whose most recent activity was more than 1000 years before present and was of dacitic magmas (Hall et al. 2017).

## Materials and methods

Sedimentary sequences 924 and 404 cm long were extracted from the deepest points of Lagunas Pindo and Baños, respectively, using a cam-modified Livingstone piston corer (Livingstone 1955; Colinvaux et al. 1999). Fourteen samples were selected from both cores and sent to the NERC Radiocarbon Facility, SUERC, East Kilbride, Scotland for radiocarbon analsysis by accelerator mass spectrometery (AMS) (Table 1). All samples underwent chemical pre-treatment to remove extraneous, contaminant carbon; full details of sample pre-treatment and conversion to graphite can be found in ESM2. Calibration was made with CALIB 7.0.4, and the IntCal13.14c database and SHCal.13.14c for Lagunas Baños and Pindo respectively (http://calib.qub.ac.uk./calib/, last accessed October 2015). Age-depth models were constructed using the statistical package clam in R (Blaauw 2010).

**Table 1:** Conventional (yBP) and calibrated (cal yBP) Radiocarbon data used in construction of chronologies for Laguna Baños and Laguna Pindo

Publication code	Depth (cm)	δ^13^C_VPDB_ (‰)*	^14^C age (yBP)	Calander age (cal year BP) 2σ	Median age (cal year BP)^a^
*Laguna Baños*					
SUERC-50081^b^	24	− 26.4	1219 ± 35	1061–1189	1090
SUERC-54389^b^	40	− 24.7	1364 ± 41	1236–1346	1288
SUERC-43521^b^	63	− 26.0	1457 ± 36	1299–1402	1357
SUERC-43524^b^	98	− 23.5	1497 ± 38	1307–1420	1400
SUERC-43525^b^	202	− 26.7	1721 ± 38	1552–1711	1615
SUERC-54393^b^	312	− 25.3	3530 ± 42	3694–3920	3923
SUERC-50084^b^	356	− 25.3	4308 ± 37	4832–4964	4887
SUERC-43526^b^	402	− 27.3	5785 ± 39	6491–6671	6583
*Laguna Pindo*					
SUERC-54395^c^	46	− 30.2	334 ± 42	289–470	375
SUERC-47634^c^	117	− 27.9	974 ± 36	769–923	849
SUERC-47635^c^	245	− 27.3	1973 ± 39	1812–1943	1878
SUERC-47569^c^	329	− 24.9	2335 ± 37	2293–2361	2283
SUERC-47572^c^	410	− 22.7	2829 ± 39	2781–2991	2916
SUERC-48854^b^	461	− 28.7	3974 ± 45	4241–4447	4342

* δ^13^C values were measured on a dual inlet stable isotope mass spectrometer (Thermo Scientific Delta V Plus) and are representative of δ^13^C in the pre-treated sample material

^a^Weighted average

^b^Bulk sediment samples

^c^Wood remains’ samples

**Fig. 2:**
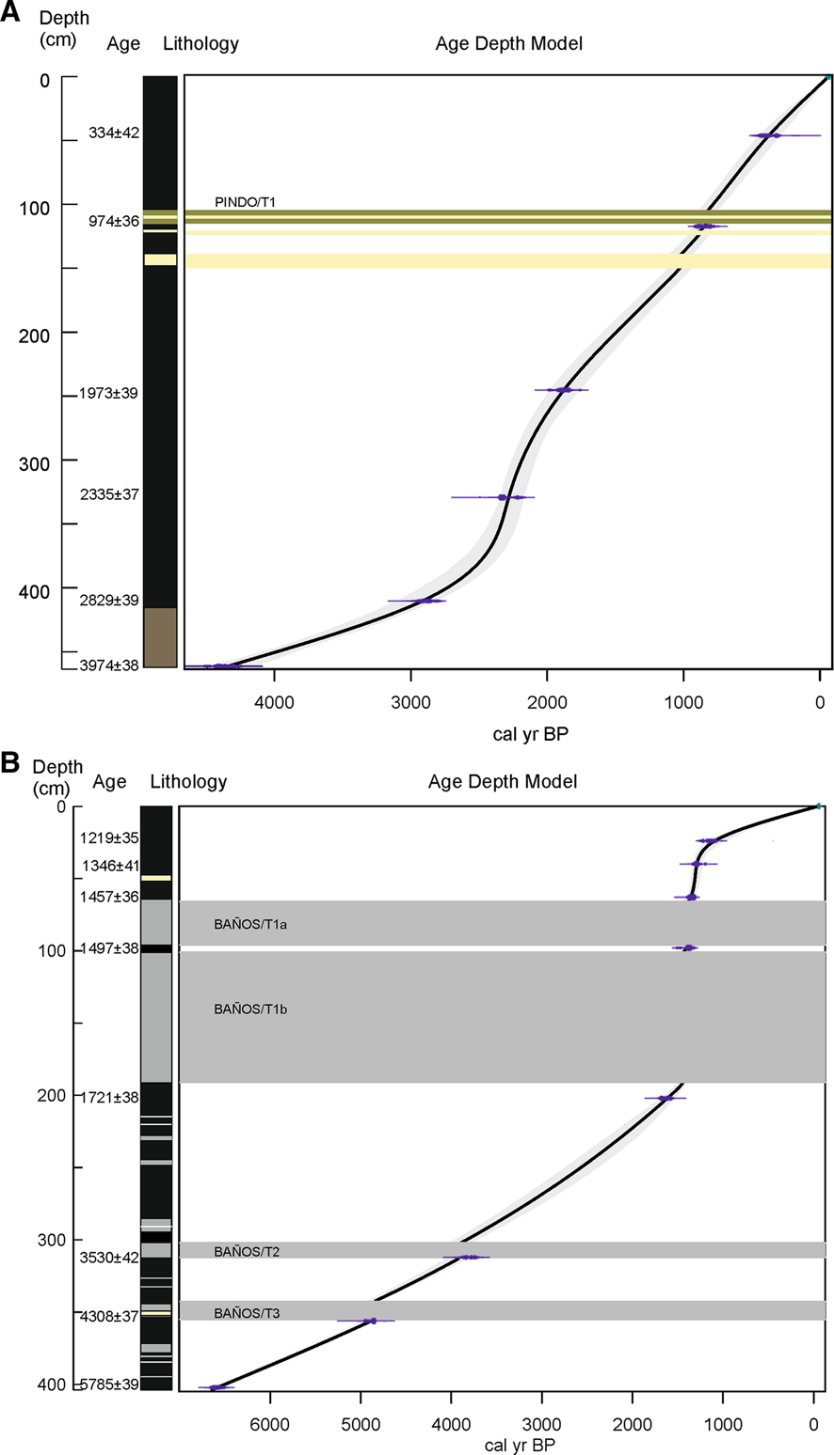
Sediment lithology, radiocarbon dates (uncalibrated age), position of the tephras analysed (except PINDO/B) and age-depth models of **a** Laguna Pindo and **b** Pond Baños. Key colour for sediment descriptions in the online version: Black or dark brown = organic rich sediments (peat, clay); white = grey sandy intervals; grey = compacted grey clay sediments (tephra); green = greenish sandy clay, not compacted; yellow = sediment gap (no sediment)

The composition of five tephra samples (one for L. Pindo and four for L. Baños) were determined using X-Ray Fluorescence (XRF) Elemental determinations. XRF analysis was performed with an ARL 8420+ dual goniometer wavelength dispersive XRF spectrometer at The Open University, UK. Analysis was run for major elements composition (SiO_2_, TiO_2_, Al_2_O_3_, Fe_2_O_3_, MnO, CaO, Na_2_O, K_2_O and P_2_O_5_). Samples were recovered from the main inorganic layers found in both records. One tephra deposit was identified in Laguna Pindo (105–114 cm depth). The deposit had sandy texture, a greenish colour and included some decomposed and reworked plant material. Additionally, a sample from the inorganic sediment at the bottom of the sequence was also analysed (900 cm depth, not included in Fig. 2). Samples for XRF analysis from Laguna Baños were taken from thick tephra-like deposits (Table 2), corresponding to four intervals of highly compacted grey sediments of more than 5 cm thickness (Fig. 2).

**Table 2:** Description of inorganic sediments sampled for XRF analysis and chemical composition based on the results obtained for the major elements (expressed in wt%)

Sample	PINDO/T1	PINDOB	BAÑOS/T1a	BAÑOS/T1b	BAÑOS/T2	BAÑOS/T3
Sequence	Laguna Pindo	Laguna Pindo	Laguna Baños	Laguna Baños	Laguna Baños	Laguna Baños
Depth	105 cm	887 cm	93 cm	161 cm	307 cm	348 cm
Tephra thickness	105–114 cm	861–924 cm	66–96 cm	101–191 cm	302–312 cm	342.5–355 cm
Age (cal year BP)	825	> 50,000	1380	1470	3600	4625
Colour	2.5 YR—6/4	2.5 YR—6/1	10 YR—5/1	10 YR—5/1	10 YR—6/2	10 YR—5/1
Texture	Sandy-silty sediment	Sandy clay	Clay	Clay	Clay	Sandy clay
Compaction	Not very compacted; reworked plant material	Highly compacted	Highly compacted	Highly compacted	Highly compacted	Highly compacted
SiO_2_	64.7	45.79	68.33	67.47	64.51	60.73
TiO_2_	0.31	1.814	0.295	0.31	0.356	0.516
Al_2_O_3_	15.46	36.76	14.9	15.31	16.14	17.36
Fe_2_O_3_	2.32	1.26	2.73	2.86	4.01	5.36
MnO	0.054	0.008	0.06	0.061	0.075	0.093
MgO	1.67	0.09	1.59	1.74	2.01	2.61
CaO	2.99	0.11	2.81	3.15	4.34	5.99
Na_2_O	4.24	0.05	4.4	4.6	4.33	4.08
K_2_O	1.93	0.14	2.26	2.18	1.29	1.22
P_2_O_5_	0.189	0.104	0.115	0.124	0.159	0.149
LOI	6.16	14.32	2.08	1.84	2.6	1.29

Age expressed in calendar years before present (cal year BP). Colour has been estimated using Munsell Color Chart

*LOI* loss of ignition

A total of 56 samples 0.5 cm wide from both cores were analysed for chironomid remains, 32 samples at 10–16 cm intervals from Laguna Pindo, and 25 samples at 8–16 cm intervals from L. Baños. Analysis of sub-fossil chironomid larval head capsules followed standard methods (Brooks et al. 1997). Head capsules were dehydrated in 100% ethanol and mounted in Euparal dorsal side up. Specimens were identified to the highest taxonomic resolution under a light microscope with reference to Wiederholm (1983), Brooks et al. (2007), and local taxonomic works including Prat et al. (2011), Trivinho-Strixino (2011), Williams et al. (2012) and Matthews-Bird et al. (2016b). Diagrams were plotted with PSIMPOLL 4.27 (Bennett 2009) and zonations were performed by “Optimal Splitting by Information Content” (OSIC), using the broken stick method to determine the significant zones (Bennett 1996).

Remains of aquatic and littoral vegetation, including aquatic and semi-aquatic pollen grains, fern spores, and algal and zoological remains other than chironomids, were also analysed. 1 cc of wet sediment was processed from each of 63 samples (29 from Laguna Pindo, 10–20 cm sampling interval; and 34 from Baños, 10 cm interval), following standard palynological protocols (Faegri and Iversen 1989). Slides were mounted in glycerine jelly. Taxa abundances were expressed as percentage with respect to terrestrial pollen taxon sum. Identification was made according to Colinvaux et al. (1999), Hooghiemstra (1984), and Hooghiemstra and van Geel (1998). Diagrams were plotted with PSIMPOLL 4.27 (Bennett 2009) and the zones shown are the same as those used in the chironomid diagrams.

Detrended correspondence analysis (DCA) was performed using square root transformed percentage species assemblage data, as an indirect ordination method to assess the variation in species as compositional units of turnover. Statistical analysis was carried out in R, using the package Vegan (Oksanen et al. 2013).

## Results

### Chronology

The best-fit age-depth model for L. Pindo was a smooth spline (Fig. 2). Six radiocarbon samples were used to date the sequence to 416 cm. Below 416 cm chironomid head capsules were absent. The sedimentation rate ranged between 0.03 and 0.5 cm year^−1^, giving an average sampling interval of 97 years (range from 26 to 197 years). Average sampling resolution for the rest of the biological proxies was 99 years, ranging from 16 to 290 years.

Eight radiocarbon samples from L. Baños were used to produce a smooth spline age-depth model for the entire sequence (Fig. 2). The sedimentation rate ranged between 0.017 and 0.5 cm year^−1^, giving an average sampling interval of 282 years (range from 19 to 608 years). There was a marked variability in the sedimentation rate between the top and bottom sections of the Laguna Baños sequence that has resulted in different analysis resolution, due to sampling was carried out based on equidistant depths. In this sense, the average sedimentation rate of the upper (younger) 300 cm of the record resulted in around 200 years per sampling interval, whilst the resolution achieved in the bottom (older) 100 cm was almost of 500 years. Average sampling resolution for the rest of the biological proxies analysed was 214 years between samples, ranging from 24 to 565 years.

### Tephras

Tephra is a much more common feature within the sedimentary sequence of L. Baños than in Laguna Pindo. In L. Baños, at least four large layers (> 5 cm thick) have been identified as possible tephra deposits whereas just one can be clearly observed in Laguna Pindo (Fig. 2).

**Fig. 3:**
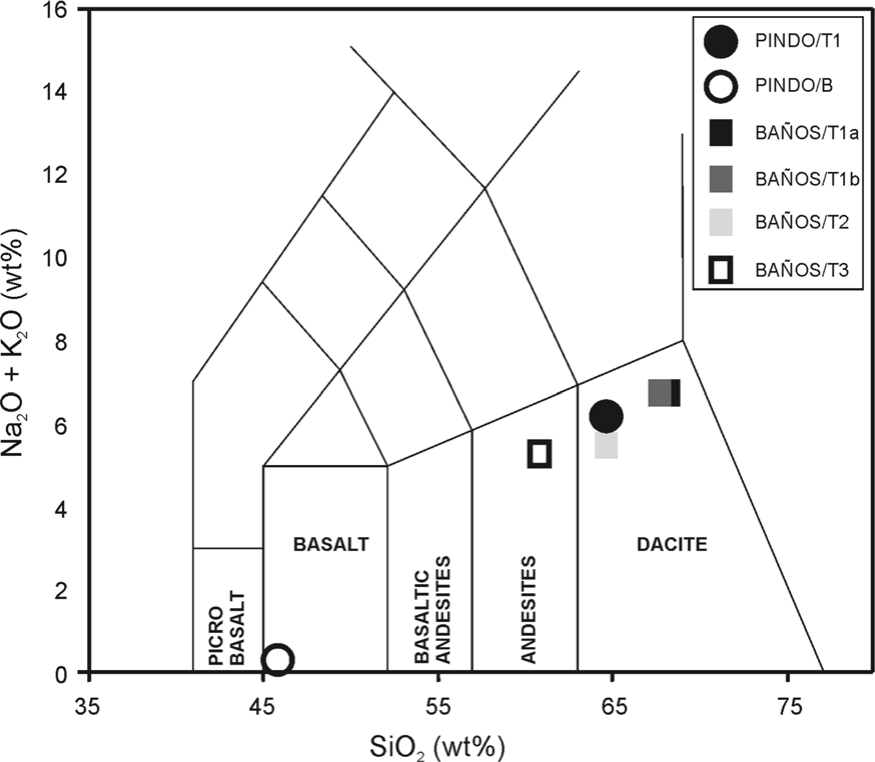
Total alkali–silica (TAS) plot of the considered tephras following classification of Le Bas et al. (1986), and based on the results of Table 2

XRF analysis of tephras shows a similar composition in major elements for all samples apart from the basal sample of L. Pindo (Sample PINDO B; Table 2), which is not a tephra. This can also be seen in the total alkali-silica (TAS) plot (Fig. 3). BAÑOS T1a and T1b are plotted together in the upper-right, to the left is the only tephra found in L. Pindo (PINDO T1) and BAÑOS T2. All these samples are located within the dacite-composed domain. BAÑOS T3 is associated with the andesite-domain. PINDO/B is unlike any other sample and belongs to the basalt-domain. This result suggests the likely tectonic origin of the lake. Based on the XRF data (Table 2 and ESM3), ^14^C dating chronology (Fig. 2) and visual examination of minerals contained in the inorganic sediments analysed (ESM4), we suggest that tephras BAÑOS T1a and BAÑOS T1b are from the same volcanic eruption (called BAÑOS T1), BAÑOS T1b being the original deposition and BAÑOS T1a a further input caused by either sediment collapse of the surrounding slopes or sediment arrival by inflow of upstream waters. The potential origin of tephra BAÑOS T1 could be from volcano Antisana (Hall et al. 2017), although other sources from the same volcanic district should not be ruled out (EMS3). Regarding the tephra found in Laguna Pindo (PINDO T1), a plausible source could be Tungurahua based on proximity and volcano activity. However, the dacitic nature of the ashes and the radiocarbon date obtained in the organic sediment below (117 cm), also supports a likely origin from the Quilotoa great eruption 800 cal year BP ago (Mothes and Hall 2008). Nevertheless, the suggestions about volcano sources of the tephras found are very preliminary and further analyses are required to confirm or reject both hypotheses.

### Chironomids

#### Laguna Pindo

Chironomid remains were only found in the upper 414 cm (corresponding to the 32 samples used for this work) of the entire 924 cm sequence of Laguna Pindo. In total, 2461 individual chironomid head capsules (hc) were analysed. The entire assemblage included 45 taxa in 26 genera and 3 subfamilies. Among the taxa identified, 27 were Chironominae, 17 were Orthocladiinae and there was one Tanypodinae. Average head capsule concentration of samples between 0 and 200 cm (last 1500 year) was 106 hc g^−1^, compared with 44 hc g^−1^ from sediments below 200 cm (older than 1500 year BP).

**Fig. 4:**
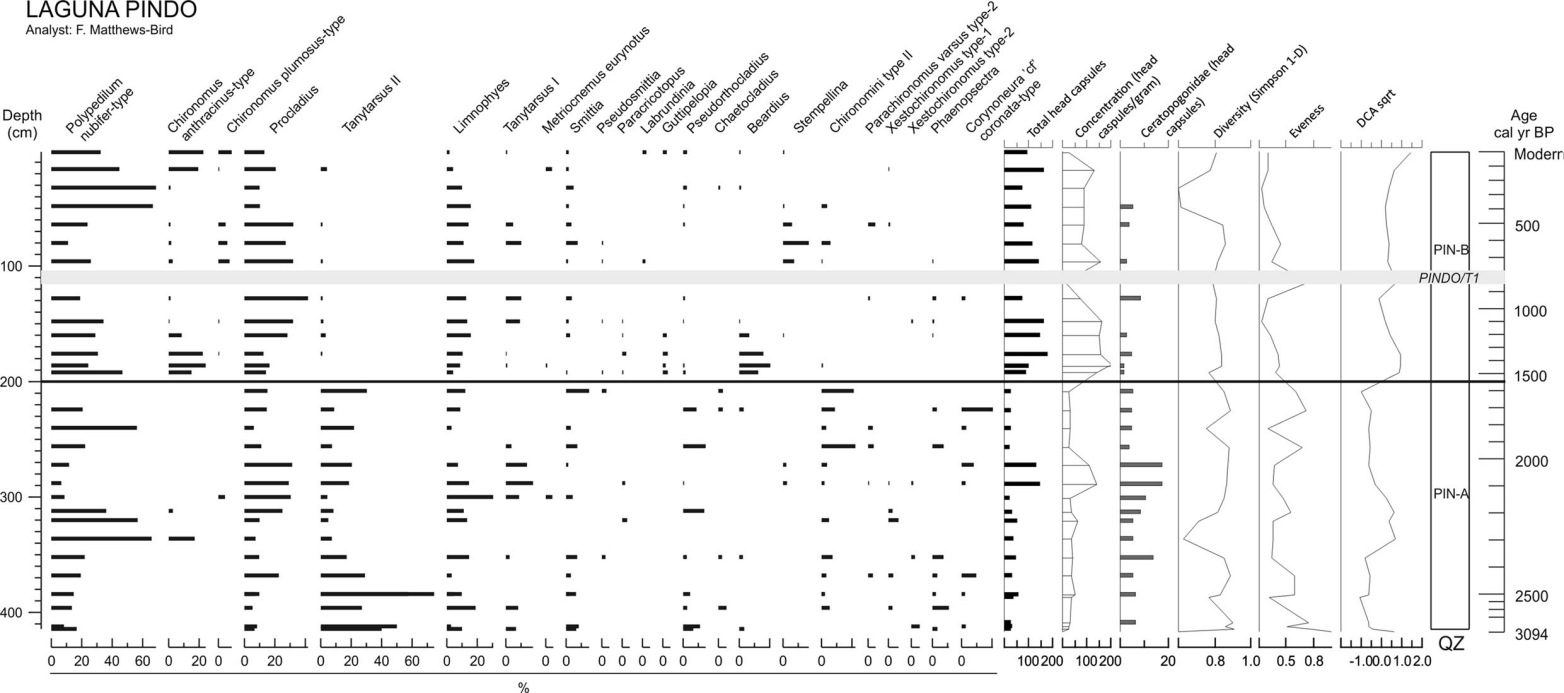
Percentage diagram of chironomids in Laguna Pindo. QZ: Chironomids zones. The grey band denotes tephra deposition (labelled in italics). DCA axis score was calculated with chironomid abundance square root transformed

*Polypedilum nubifer*-type, *Procladius* and *Limnophyes* were the most abundant taxa throughout the record (> 10% wherever they occurred) (Fig. 4). Note the relationship in the observed trends between *Polypedilum nubifer*-type and *Procladius* and *Tanytarsus* I through the record. Based on major changes in chironomid assemblage composition revealed by zonation and DCA axis 1 scores, two significant zones were identified.

Zone PIN-A (200–414 cm) is characterised by a low concentration of hc and high abundance of Ceratopogonidae. *Tanytarsus* II is present throughout the zone but is at highest abundance in the lowermost part of the zone. The middle section is characterised by a decrease in *Tanytarsus* II, increase in *Polypedilum nubifer*-type and the first appearance in the record of *Chironomus anthracinus*-type. This is shortly replaced by an increased abundance of *Procladius* and *Limnophyes*. The uppermost part of this zone is dominated by *Polypedilum nubifer*-type, *Tanytarsus*-II, and Chironomini type II. The highest abundance of *Corynoneura* cf. *coronata*-type is observed in this upper section too.

In Zone PIN-B (0–200 cm), the chironomid community shows a substantial change in its dominant taxa, with a dramatic decrease in *Tanytarsus* II and the appearance in a high abundance of other taxa barely represented in the previous zone, such as *Chironomus anthracinus*-type and *Ch. plumosus*-type. Compared to PIN-A, concentrations of chironomid hc and Ceratopogonidae are higher and lower respectively in this zone. *Polypedilum nubifer*-type, *Ch. anthracinus*-type and *Beardius* (absent from the majority of the sequence), peak around 160–200 cm (20%), immediately after the beginning of the zone. The abundance of these last two taxa shortly declines and the upper section, before the tephra PINDO/T1, is dominated by *Procladius*, *Limnophyes*, and *Tanytarsus* I. This dominance continues after the tephra, together with the appearance of other taxa, although in less abundance, such as *Ch. plumosus*-type, *Stempellina*, and Chironomini type II. The upper section of the record is characterised by a generally decreasing trend in many taxa, except for *P. nubifer*-type and *Procladius*, and during the uppermost 20 cm, *Ch. anthracinus*-type and *Ch. plumosus*-type.

#### Laguna Baños

In total 725 chironomid hc were analysed from the sediments of L. Baños. The assemblage was made up of 18 different taxa in 4 sub-families: 7 Chironominae, 10 Orthocladiinae, and one Tanypodinae. Chironomid abundance varied throughout the sequence (average: 30 hc per sample; range: 1 or 2–91 hc) as did concentration (average: 24 hc per gram; range: 1–69 hc); Ceratapogonidae was present in only two samples in the entire record. The three most dominant taxa throughout the record were *Polypedilum nubifer* type, *Pseudochironomus* and *Cricotopus/Paratrichocladius* type-II. Based on zonation analysis, two significant zones were established, separated by a large inorganic deposit (BAÑOS T1).

**Fig. 5:**
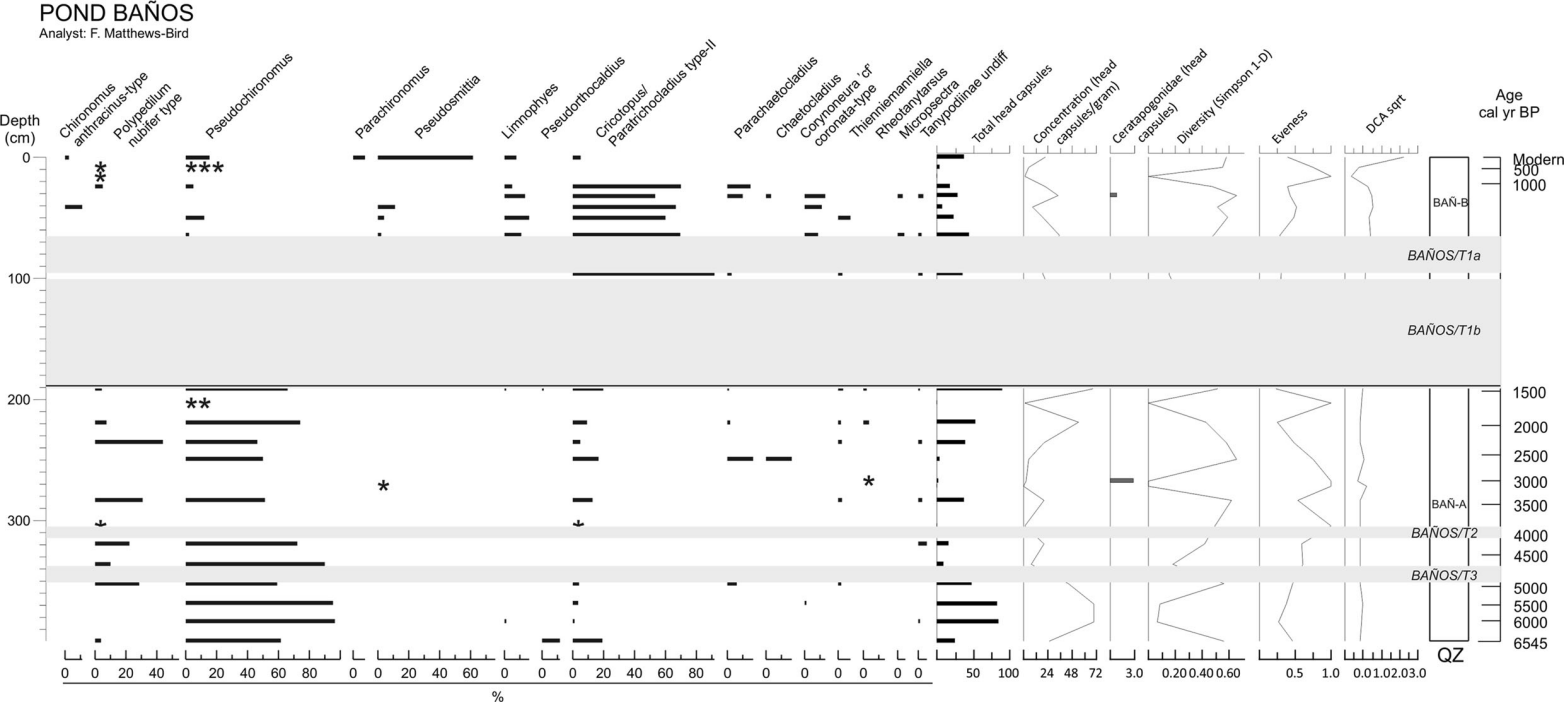
Percentage diagram of chironomids in Laguna Baños. QZ: Chironomids zones. The grey bands denote tephra depositions (labelled in italics). DCA axis score was calculated with chironomid abundance square root transformed. Asterisks mark presence of taxa in samples with very low concentration of head capsules (samples with < 10 head capsules in total)

In Zone BAÑ-A (191–404 cm), *Pseudochironomus* dominates the assemblage (Fig. 5). The lowermost sample is also characterised by low abundances of *Pseudorthocladius* and *Cricotopus/Paratrichocladius* type-II. During intervals where *Pseudochironomus* slightly decreases in abundance, other Chironomini, such as *Polypedilum nubifer* type and *Cricotopus/Paratrichocladius* type-II, increase. All other taxa occur only intermittently, including lentic morphotypes, such as *Parachaetocladius*, *Thienemanniella* and *Rheotanytarsus*, especially from around 270 cm upwards. The occurrence of taxa indicative of flowing waters coincides with low counts, low diversity and high evenness.

Zone BAÑ-B (0–191 cm): Within the 90 cm deposits of tephra BAÑOS T1b and the 30 cm of T1a no chironomid remains were found. Above the tephra deposition the chironomid community is dominated by a completely different assemblage than previously (Fig. 5). The previous zone is dominated by Chironomini which is replaced by Orthocladiinae. *Cricotopus/Paratrichocladius* type-II, *Limnophyes* and *Corynoneura* cf. *coronata*-type are the most abundant taxa in this zone. *Cricotopus/Paratrichocladius* type-II is present in small numbers in earlier sediments (Zone BAÑ-A) but is present in high abundance after the tephra deposition. However, in the samples between 24 and 8 cm some components of the pre-eruption community partially return, although with very low values (Fig. 5). This assemblage pattern is also visible in the DCA biplot (ESM5). Sample 0 (uppermost sample) represents the modern assemblage and includes taxa such as *Pseudosmittia* and *Parachironomus,* not present in the assemblage before. This sample plots away from all other samples suggesting a different composition to anything previously recorded in the sequence.

### Other aquatic biological proxies

**Fig. 6:**
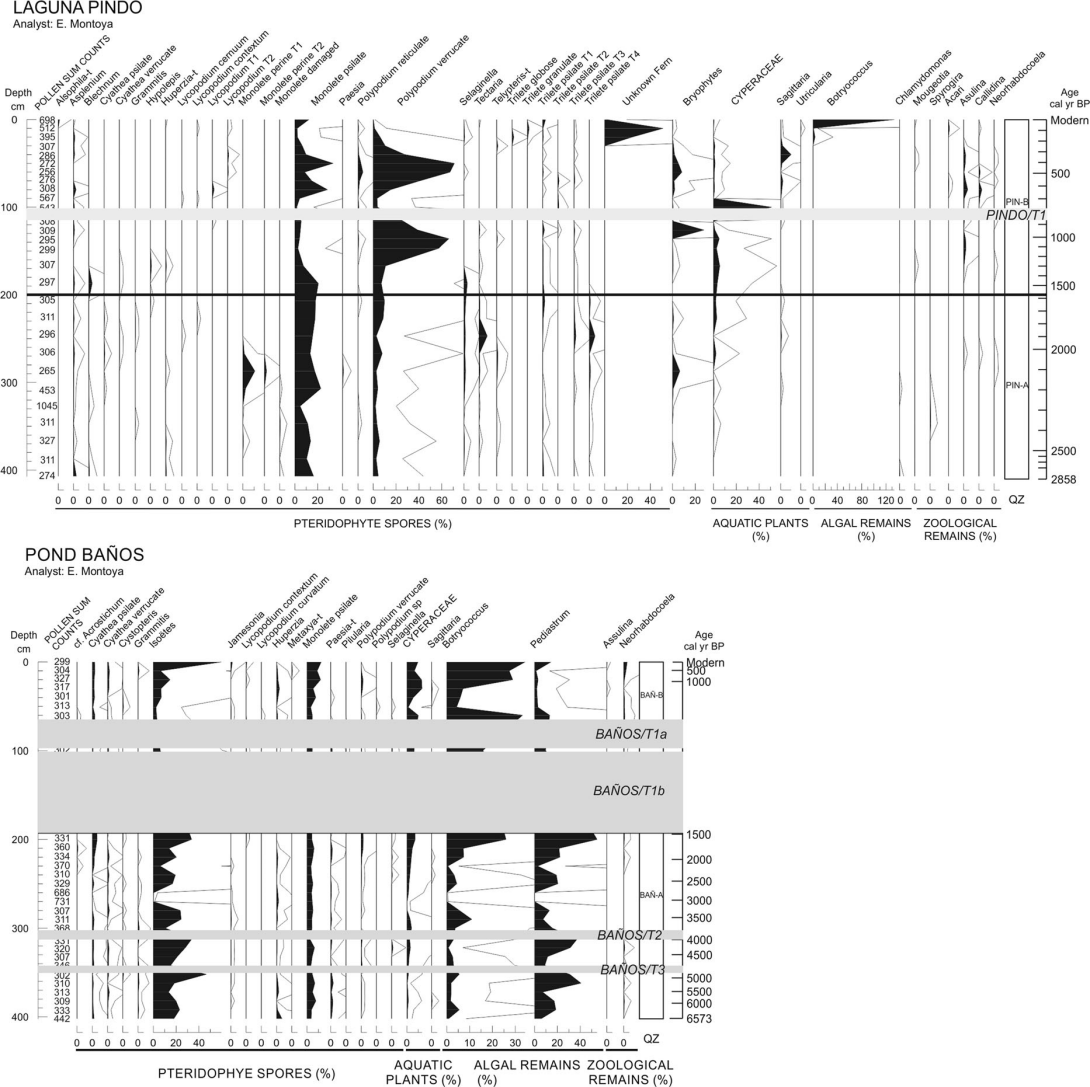
Percentage diagrams (based on the sum of total terrestrial pollen, counts shown in the first column) of other aquatic communities (ferns, aquatic and semiaquatic plants, algae and other zoological remains other than chironomids) of the two sedimentary sequences studied. Grey bands indicate tephra deposits (labelled in italics)

The Laguna Pindo record is dominated by spores in zone PIN-A (Fig. 6), Monolete psilate being the most abundant taxon, and sporadic peaks of ferns, such as Monelete perine (T1 and T2), *Tectaria* and Trilete psilate T4. *Polypodium* verrucate have a continuous presence throughout the zone but with low abundances and bryophyte spores peak at the top of the zone (Fig. 6). PIN-B is characterised at first by an increase in Cyperaceae, that began to increase near the top of the previous zone, and a marked decrease in monolete psilate and a large increase in *Polypodium* verrucate. After the tephra deposit fern spores decrease in abundance and there is a peak in Cyperaceae abundance, which then rapidly returns to low values attained in zone PIN-A. Following this peak, there are increases in monolete psilate, *Polypodium* verrucate, Bryophytes and *Sagittaria* sp. The top of the zone is marked by the almost disappearance of *Polypodium* verrucate, and the sudden increase of an unknown fern and *Botryococcus* sp.

The sequence from L. Baños is also characterised by a dynamic record despite the lower number of taxa found. The first zone (BAÑ-A) is dominated by the aquatic fern *Isoëtes* sp. and the alga *Pediastrum* sp., followed by Monolete psilate, and *Botryococcus* to a lesser extent. The dominance of *Isoëtes* and *Pediastrum* in this zone is interrupted by the tephra deposits (Tephras BAÑOST2 and T3). Nevertheless, both taxa recover to former values after the inorganic deposits. Cyperaceae shows an increasing trend through this zone. Zone BAÑ-B shows a change in the community assemblage. *Botryococcus*, Cyperaceae and monolete psilate are the dominant elements, whereas *Pediastrum* and *Isoëtes* are present in lower abundances than in BAÑ-A. *Neorhabdocoela* sp. oocytes are also more abundant in this upper zone. The top sample is characterised by peaks in *Botryococcus* and *Isoëtes*, and, to a minor extent, *Pediastrum*, which show an increasing trend through the zone.

## Discussion

### Chemical effects

The most conspicuous change in the aquatic community of the studied lakes was in L. Baños after the deposition of tephra BAÑOS T1. Pre-eruption assemblages (Zone BAÑ-A) are dominated by *Pseudochironomus* whereas post-eruption assemblages are dominated by *Cricotopus/Paratrichocladius* type-II (Zone BAÑ-B). This taxon appears in high abundance even in the brief period between ash deposits BAÑOS T1a and T1b (Fig. 5). This result reflects findings in other records. For instance, Heinrichs et al. (1999) noted an increase in *Cricotopus/Orthocladius* after deposition of the Mazama ash in Lake Kilpoola, British Columbia, whilst Araneda et al. (2007) noted a decrease in *Pseudochironomus* and an increase in *Cricotopus/Orthocladius* within the deposits of the 1956–1957 Llaima volcanic eruptions in Lake Galletue, Chile. These authors interpreted the changes to be the result of increased salinity caused by the volcanic deposition. Chemical weathering involves the solution of silica through leaching (Telford et al. 2004). Despite these similarities, our data differ from previous works as follows: i) the dominance of these taxa in previous studies was a short-lived feature (up to subcentennial), whereas in our record *Cricotopus/Paratrichocladius*-II dominates the assemblage for a longer period (around 380 years) after the ash deposit; and ii) the accumulation of the tephra causing those changes is much thicker in Baños (BAÑOS T1b: 90 cm + BAÑOS T1a: 30 cm) than in other records (< 10 cm, except for one tephra in Heinrichs et al. (1999) of around 30 cm thick) (Araneda et al. 2007; Telford et al. 2004). Such dominant deposits of tephra may not only directly affect the aquatic communities but may also cause instability and erosion of underlying mineral horizons in the catchment (Heinrichs et al. 1999). In this way, increased weathering from tephra deposition could lead to more Si being leached into the lake (Shoji et al. 1981; Telford et al. 2004), and therefore promote a sustained increase in salinity from increased erosion of mineral material over a long period. However, complementary analysis of another proxy sensitive to salinity, such as diatoms, would be essential to confirm this hypothesis. Michelutti et al. (2016) performed palaeolimnological analyses focused on diatom and chironomid assemblages in the most upstream water body of the Lake Baños system (Fig. 1) and showed that a volcanic ash deposit, likely corresponding to Tephra BAÑOS T1a, caused a spike in Hg concentration. This upstream Baños record did not extend prior to the large eruption, so comparisons between pre- and post-eruption assemblages or testing the salinity hypothesis remain elusive. However, field measures of conductivity differ in these two close water bodies, being upstream 58 µS cm^−1^(Michelutti et al. 2016) and downstream 194 µS cm^−1^.

Alternatively, changes in chemistry following tephra deposition could be related to water acidification. Self et al. (2015) analysed the effect of Holocene volcanism in central Kamchatka (Beringia) based on pollen, diatom and chironomid analyses. The authors observed that richness of diatoms and chironomid species increased when pH increased during quiescent periods without volcanic activity, and suggested that lake biota were primarily responding to water chemistry changes driven by tephra impacts, whereas catchment vegetation was primarily responding to climate (Self et al. 2015).

### Physical effects

Laguna Baños has more numerous and thicker tephra deposits than L. Pindo. The thicker deposits have a greatest impact upon chironomid communities, although the thinner deposits do result in subtle changes in evenness and diversity (tephras BAÑOS T2 and BAÑOS T3; Fig. 5). Accordingly to the minor tephra layers in Baños, there is no indication of a major shift in chironomid assemblages after the eruption at Laguna Pindo either (Zone PIN-B; Fig. 4). At L. Baños a change in the assemblages of other proxies before and after the deposition of the tephra BAÑOS T1 (Fig. 6) is also apparent. The littoral plant community occurring before the BAÑOS T1 deposit is characterised by taxa indicative of clean, oligotrophic waters, and the high abundance of the aquatic fern *Isoëtes* suggests the lake was relatively deep (Gosling et al. 2008). In the upper section of the record (after the tephra deposition) a decrease in those taxa and an increase in generalist algae (including those occurring in eutrophic waters) and Cyperaceae, suggest that the lake has become shallower (Gosling et al. 2008; Montoya et al. 2010). In Laguna Pindo, the only marked change observed in the aquatic vegetation is the sudden peak of Cyperaceae just after the tephra deposit, suggesting an ephemeral increase in the littoral zone or opening of the terrestrial vegetation. However, in this case the taxon returned to previous abundance in the next sample above.

Moreover, in Laguna Pindo the sediment associated with the tephra layer was characterised by a non-compacted material and contained some chironomid remains. BAÑOS T1, by comparison, is composed of very compacted sediment and no chironomid remains. In the study made in the L. Baños system by Michelutti et al. (2016), the post-tephra chironomid community was similar to the one we observed. The authors also highlighted the thickness and compaction of the ash deposit found. Such a compact, indurated deposit prevented the recovery of the full length of the tephra and the sediment below it, as well as the study of the aquatic communities present in the water body prior the tephra event (Michelutti et al. 2016). This compacted layer would have buried the benthic communities and limited bioturbation. In our data, the only observed change in pelagic autotrophic organisms corresponds to the decrease in *Pediastrum* observed in the upper section of L. Baños (Fig. 6). The results suggest that the effect of the tephra on the chironomid community is likely physical, as the thickest tephra deposit has greatest impact. This suggestion is not just based on the comparison between the most recent tephras at Pindo and Baños, but also between different tephras at Baños, where the thinner tephra layers have less impact even though they have similar chemical composition (Table 2 and ESM3; Fig. 3). Both chironomids and other aquatic organisms show a similar response, in which the older (thinner) tephras at Baños caused negligible impact on the community but the large tephra deposit resulted in a regime shift, indicated by a marked and sustained assemblage turnover. Based on the available evidence presented here, we suggest that this shift is related to a substantial shallowing of the lake produced by the deposition of a > 1 m thick ash layer, which considering that the lake is today about 1 m deep, could have potentially halved the depth of the lake at the time of the deposition. This hypothesis is supported by the increase of littoral Orthocladiinae taxa in Zone BAÑ-B. Thus, the profound change in the bathymetry of the water body basin is a major driver of the long-term assemblage shift, causing a loss of resilience of the community after a threshold event (Seddon et al. 2014; Hodgson et al. 2015).

The recovery of such a thick volcanic deposit as BAÑOS T1 in a sedimentary archive is unusual. As already mentioned, most of the previous studies analyse the effects of thin tephras upon the catchment or lake system, and any changes reported in the biota are often short-term. Besides burial, the immediate consequence to aquatic communities of tephra deposition is reduced light availability which is detrimental to aquatic macrophytes and photosynthetic algae (Abella 1988). Tephra settling velocity, however, is fast (> 40 m h^−1^) and is unlikely to account for ecological responses of > 1 year (Telford et al. 2004). None of the present records have provided evidence to support light suppression of the aquatic flora following the ash deposition (Fig. 6), maybe because the temporal resolution is too coarse considering the rapid biological cycle of the pelagic algal taxa involved.

### Catchment features’ effects

The two sites in our study have very different catchments. Laguna Pindo is completely vegetated with dense canopy forest and no river inflow. In contrast, Laguna Baños is in an open grassland landscape with areas of bare rock and is the last in a series of water bodies connected by a stream (Fig. 1). The exposed landscape around L. Baños is perhaps the reason it seems to be much more sensitive to the effects of a volcanic eruption. The tephra deposits in Baños are more numerous and larger than in Laguna Pindo probably because more material can enter the lake due to the open landscape and the cascading effect of being downstream of tephra sinks. On the other hand, the lower amount of tephra deposited in PINDO T1 would have been influenced by the nearness of erupting dacitic volcanoes and the physical barrier of the forested surroundings (Fig. 1). Related to this could also be the different provenance of the volcanic deposits. Whereas Laguna Pindo only received one thin tephra, L. Baños received many volcanic deposits, coming potentially from at least two different sources (ESM4). Besides vegetation cover, wind direction is a major driver in the amount of ash arriving in both landscapes, but also L. Baños is located nearer to large young dacite eruptive centers such as Antisana and Guagua Pichincha (Hall et al. 2008, 2017), “Cosanga volcanoes”. On the other hand, as L. Baños is river-fed, the lake can receive ash over long periods from the catchment, whereas L. Pindo is fed only by precipitation. This is reflected in the sensitivity of the chironomid assemblages of the Laguna Baños system, in which the upstream assemblage is more stable (Michelutti et al. 2016) than downstream assemblage.

Finally, it is also important to consider human land-use practices altering the surrounding landscape, which may increase the lake’s vulnerability to an eruption. Humans have occupied the Andes since the early Holocene (Rademaker et al. 2014), however, evidence for sustained and high intensity manipulation of the landscape does not appear until after 4000–3000 cal year BP (Chepstow-Lusty et al. 2011). The modern treeline occurs around 3200 m a.s.l., however, in the absence of humans, it is thought to lie closer to 3900 m (Ellenberg 1958; Kessler 1995). Deforestation can modify the catchment area and therefore may increase the sensitivity of high Andean lake ecosystems to volcanic eruptions. Deforestation (and hence, erosion) facilitates the in-wash of higher amounts of ashes into the aquatic system. Dams may have similar effects by compacting and thickening the tephra held in a dammed lake. The sensitivity of high altitude lakes is particularly important, as L. Baños is located within the Cayambé Coca National Park, which is the main water supply for the Metropolitan District of Quito and surrounding areas (> 1.5 million of people) (Echavarría and Lochman 1998). Another human impact is eutrophication, which can affect water quality. The uppermost samples of L. Pindo are characterised by the presence of *Chironomus anthracinus* and *Ch. plumosus* and a decrease in *Tanytarsus*-II, and dense aquatic vegetation suggesting the potential effect of recent eutrophication. Whether the recent eutrophication is naturally forced by lake infilling or is human-induced cannot be discerned, however, without further analysis.

## Conclusions

Volcanism has been a constant feature of Quaternary environments in the tropical Andes. Combined with on-going climate change and human land use practices, however, high impact volcanic events have the potential to radically alter Andean aquatic ecosystems. Our data suggest that tephra thickness (i.e., the amount of ashes deposited within a system) is the most important factor determining the lasting impact of an eruption on the chironomid community. Tephra thickness is particularly important in shallow systems, as it will dramatically change the bathymetry and may result in an ecological threshold being crossed. The impact of tephra deposition can be magnified by catchment features such as the water bodies’ connectivity and extent of vegetation cover. Laguna Pindo, which has a small, non-connected, catchment and is heavily forested to the water’s edge, is buffered from long-term impacts caused by an eruption. Laguna Baños, in contrast, is more exposed to long-term effects of a high impact event and thus more sensitive. It is also located closer to volcanoes that produce large silica-rich ash volumes. Chemical effects, which are also dependent on the amount of tephra deposited, may also contribute to the amount of change in the aquatic communities. Finally, the intensity of human land-use practices, such as deforestation and damming, may magnify these impacts by allowing rapid deposition of large amounts of tephra and decreasing the resilience of the systems to change. A better understanding of ecosystem sensitivity and resilience to high impact events can help guide mitigation strategies and conservation policy. The palaeoecological record provides a framework for identifying key elements of ecosystems resilience. More empirical data on disturbance to aquatic communities caused by volcanic ash deposits, such as light suppression, chemical stress or physical burying, is required.

## Electronic supplementary material

Below is the link to the electronic supplementary material.

Supplementary material 1 (DOCX 4720 kb)
