# Supplementary material for: Aquatic community response to volcanic eruptions on the Ecuadorian Andean flank: evidence from the palaeoecological record
Source: J Paleolimnol. 2017 Oct 5;58(4):437–53. doi: 10.1007/s10933-017-0001-0 (PMC6959416; doi:10.1007/s10933-017-0001-0)

**ELECTRONIC SUPPLEMENTARY MATERIAL (ONLINE)**

**ESM1.** Map of northern South America showing the Northern Volcanic Zone (NVZ), in relation to the Equator. PI: Panama Isthmus. PC: Pacific Coast. Radar image courtesy of NASA/JPL-Caltech.


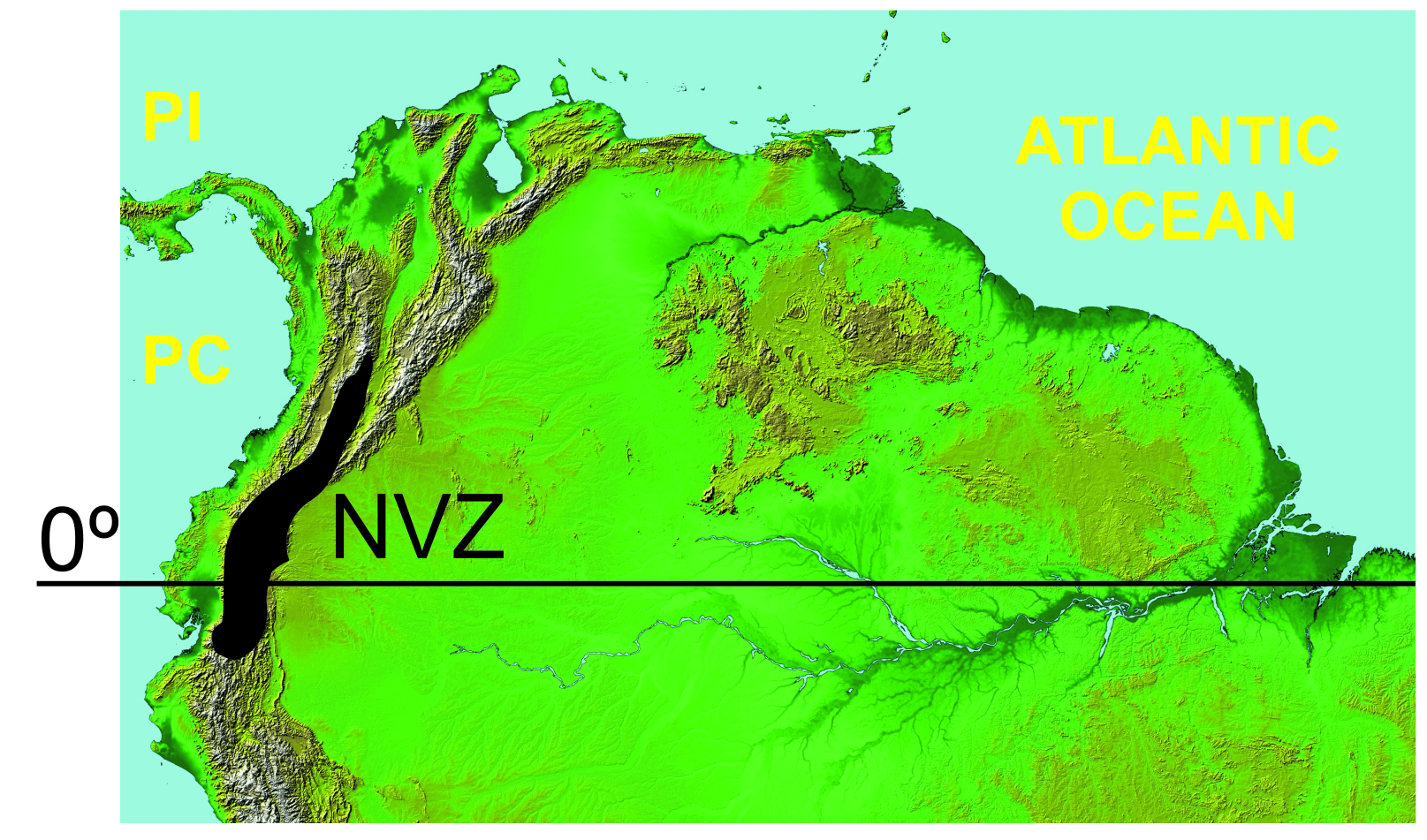


**ESM2.** Pre-treatment of samples for radiocarbon analysis.

**Bulk sediment:** Samples were acid washed in 2M HCl (8 hrs at 80 ^0^C) to remove inorganic carbon, washed free of mineral acid with distilled water, dried and homogenised.

**Wood:** Samples were broken into small pieces, digested in 4M HCl (80 ^0^C for 8 hrs), washed free of mineral acid with deionised water then digested in 2M KOH (80 ^0^C for 2 hrs). The digestion was repeated using deionised water until no further humics were extracted. The residue was washed free of alkali with deionised water, digested in 1M HCl (80 ^0^C, 5 hours) and rinsed free of acid with deionised water. The de-humified wood was digested at 70 ^0^C in acidified sodium chlorite solution (13g NaClO2 + 2ml conc. HCl in 500ml deionised water) until the entire sample had been oxidised to cellulose then filtered through glass fibre filter paper (Whatman GF/A), washed acid free with hot deionised water and dried in a freeze dryer.

The total carbon in a known weight of pre-treated sample was recovered as CO_2_ by heating with CuO and Ag foil in a sealed quartz tube. The gas was converted to graphite by Fe/Zn reduction.

**ESM3.** Chemical composition of L. Baños tephras, based on XRF analysis of trace elements (expressed in wt. %).

| **Trace Elements** | **BAÑOS T1a** | **BAÑOS T1b** | **BAÑOS T2** | **BAÑOS T3** |
| --- | --- | --- | --- | --- |
| Rb | 48 | 46 | 24 | 23 |
| Sr | 364 | 402 | 432 | 471 |
| Y | 8 | 8 | 11 | 14 |
| Zr | 65 | 62 | 96 | 86 |
| Nb | 5 | 5 | 4 | 4 |
| Ba | 801 | 789 | 610 | 584 |
| Pb | 14 | 12 | 5 | 7 |
| Th | 6 | 5 | 4 | 3 |
| U | 4 | 2 | 2 | 2 |
| Sc | 4 | 5 | 10 | 13 |
| V | 44 | 43 | 68 | 112 |
| Cr | 10 | 11 | 18 | 25 |
| Co | 6 | 6 | 9 | 14 |
| Ni | 11 | 11 | 13 | 14 |
| Cu | 47 | 39 | 25 | 22 |
| Zn | 64 | 61 | 62 | 74 |
| Ga | 17 | 18 | 17 | 18 |
| Mo | 5 | 3 | 1 | 0 |
| As | 4 | 3 | 4 | 2 |
| S | 119 | 182 | 147 | 196 |

**ESM4.** Visual examination of minerals and grain size of L. Baños tephras and hypothesis about potential volcano origin.

| **Tephra layer and depth in sediment column** | **Characteristics of tephra-mineral %, size, color, texture** | **Potential volcano** | **Supporting evidence** | **Bibliographic Reference** |
| --- | --- | --- | --- | --- |
| T1a; 93cm | Light colored, scarce small white rounded pumice- 3 %; Quartz (5%), Plagioclase- 60%; Amphibole= acicular= 5%; biotite- 10%; Vitreous glass- white-~ 15%.  Overall grain size < 100 um. Ash is very fine  Could be the windowed fines of the lower T1b layer | Cosanga Volcanoes-Pumayacu o El Dorado dome.  Ash is very acid (SiO2= 68%). | In a streamcut in Cosanga area there are layers of archeological vestiges dated at about 2000 yBP. Overlying this layer are two local-source ash layers, which are sandwiched beneath the 800 yr BP Quilotoa ash. | Hall Minard L and Mothes Patricia A. 2010. *New Active Rhyolitic Eruption Centers, Eastern Foot of the Ecuadorian Andes.*  *Abstract, Cites on Volcanoes-6, Tenerife Spain. Session:* 1.1-P-86 |
| T1b; 161 cm | Small white subangular pumice-10%; Quartz-5%; Plagioclase-60%; Black obsidian squirls- 5%, Biotite- gold and silver colored- 10%; Hornblende – 10%. Metamorphic muscovite- 1%. Overall grain size is coarser than above. Overall grain size > 150 um. | Cosanga Volcanoes- Pumayacu or El Dorado domes.  Ash is very acid (SiO2= 67%). | In a streamcut in Cosanga area there are layers of archeological vestiges dated at about 2000 yBP. Overlying this layer are two local-source ash layers, which are sandwiched beneath the 800 yr BP Quilotoa ash. | Hall Minard L and Mothes Patricia A. 2010. *New Active Rhyolitic Eruption Centers, Eastern Foot of the Ecuadorian Andes.*  *Abstract, Cites on Volcanoes-6, Tenerife Spain. Session:* 1.1-P-86 |
| T2; 307 cm | Small white subangular pumice- well vesiculated- 10%; Quartz- 3%; Plagioclase-60%; Grey smokey obsidian squirls- 5%; Hornblende- 20%; Augite- olive green color-2%; Magnetite- 5%  Overall grain size < 150 um. | Antisana volcano | A similar tephra layer (*ANTI-37*) is observed in a section NW of Antisana volcano, where it directly overlies a soil dated at 3400 +/- 150 yBP. The lower SiO_2_ (64%) is suggestive that this tephra is related to Antisana´s recent dacite activity. | (Hall et al., 2017). |
| T3; 350 cm | Small rounded White pumice- 50%: Quartz- 5%; Plagioclase- 25%, Hornblende- 10%; Black obsidian- 5%, biotite- trace; Magnetite- 5%  Overall grain size < 150 um. | Cosanga Volcanoes- Pumayacu dome | At the base of Pumayacu dome we have a dated layer of 4390 +/- 120 yBP, with a high percentage of siliceous pumice. | Hall Minard L and Mothes Patricia A. 2010. *New Active Rhyolitic Eruption Centers, Eastern Foot of the Ecuadorian Andes.*  *Abstract, Cites on Volcanoes-6, Tenerife Spain. Session:* 1.1-P-86 |

**ESM5**. DCA analysis of samples distribution based on chironomid abundances values. **a** Laguna Pindo and **b** Laguna Baños. Numbers indicate the depth of the samples in each sequence and colour defines the sample zones (white = zone PIN-A or BAÑ-A, lower zone, older samples; black = PIN-B or BAÑ-B, upper zone, younger samples).


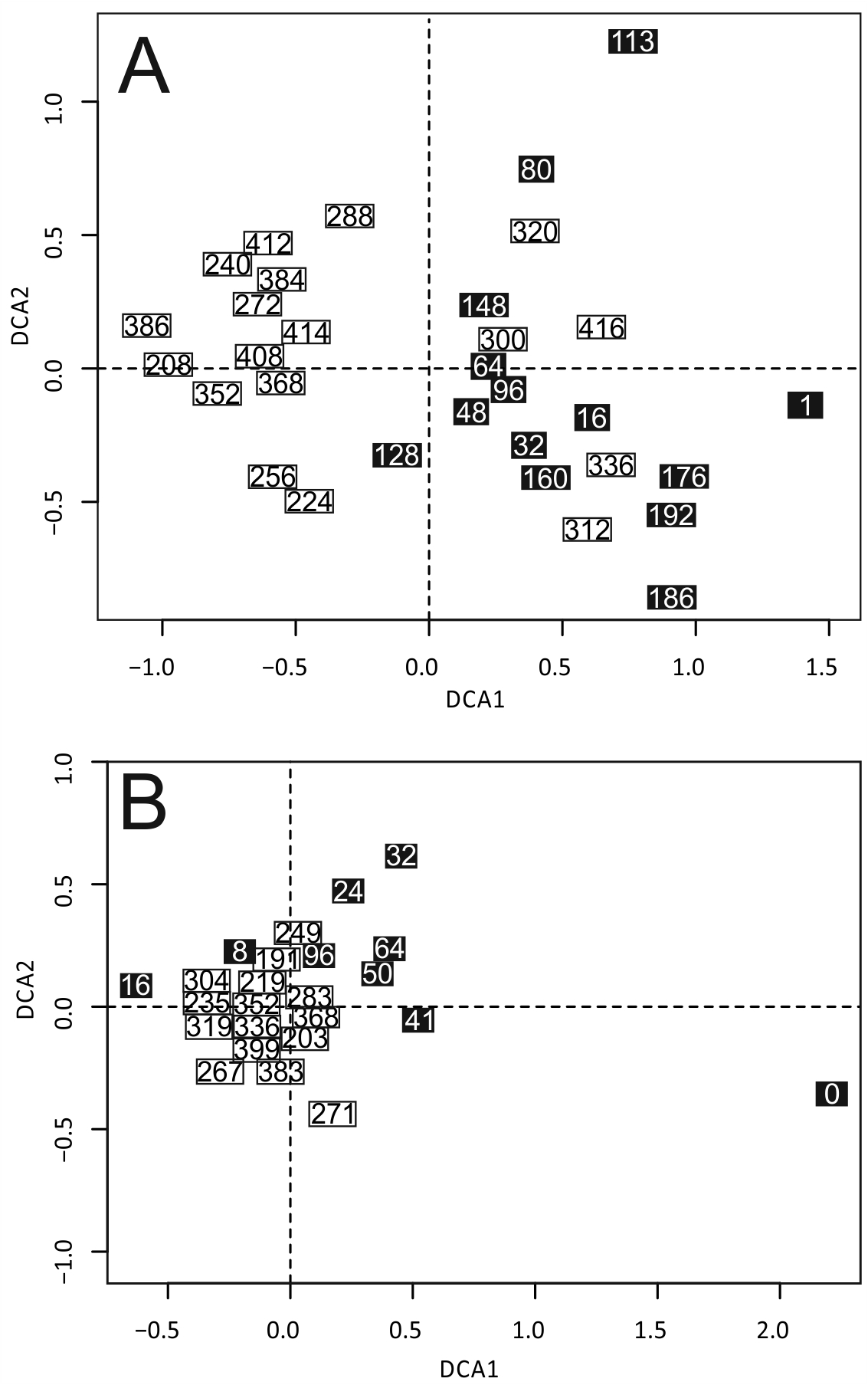

Supplement: Supplementary file 1 — Supplementary material 1 (DOCX 4720 kb) [file 10933_2017_1_MOESM1_ESM.docx]
